# Landscape of the genome and host cell response of *Mycobacterium shigaense* reveals pathogenic features

**DOI:** 10.1038/s41426-018-0116-z

**Published:** 2018-06-22

**Authors:** Haiqin Jiang, Jiya Sun, Yanqing Chen, Zhiming Chen, Le Wang, Wei Gao, Ying Shi, Wenyue Zhang, Youming Mei, Santosh Chokkakula, Varalakshmi Vissa, Taijiao Jiang, Aiping Wu, Hongsheng Wang

**Affiliations:** 1 Institute of Dermatology, Chinese Academy of Medical Sciences and Peking Union Medical College, Nanjing, 210042 China; 2 Jiangsu Key Laboratory of Molecular Biology for Skin Diseases and STIs, Nanjing, 210042 China; 3 Suzhou Institute of Systems Medicine, Center of Systems Medicine, Chinese Academy of Medical Sciences, Suzhou, 215021 China

## Abstract

A systems approach was used to explore the genome and transcriptome of *Mycobacterium shigaense*, a new opportunistic pathogen isolated from a patient with a skin infection, and the host response transcriptome was assessed using a macrophage infection model. The *M*. *shigaense* genome comprises 5,207,883 bp, with 67.2% G+C content and 5098 predicted coding genes. Evolutionarily, the bacterium belongs to a cluster in the phylogenetic tree along with three target opportunistic pathogenic strains, namely, *M*. *avium*, *M*. *triplex* and *M*. *simiae*. Potential virulence genes are indeed expressed by *M*. *shigaense* under culture conditions. Phenotypically, *M*. *shigaense* had similar infection and replication capacities in a macrophage model as the opportunistic species compared to *M*. *tuberculosis*. *M*. *shigaense* activated NF-κB, TNF, cytokines and chemokines in the host innate immune-related signaling pathways and elicited an early response shared with pathogenic bacilli except *M*. *tuberculosis*. *M*. *shigaense* upregulated specific host response genes such as *TLR7*, *CCL4* and *CXCL5*. We performed an integrated and comparative analysis of *M*. *shigaense*. Multigroup comparison indicated certain differences with typical pathogenic bacilli in terms of gene features and the macrophage response.

## Introduction

Among the >100 species described in the genus *Mycobacterium*, *M*. *tuberculosis* and *M*. *leprae* are the two most notorious agents. *M*. *tuberculosis* is responsible for millions of tuberculosis cases worldwide annually^1^, whereas *M*. *leprae* causes chronic infectious disease that can result in debilitating deformities and slowly progress throughout one’s life if left untreated^2^. In addition to *M*. *tuberculosis* and *M*. *leprae*, nontuberculous mycobacteria (NTM) can also cause pulmonary diseases that resemble tuberculosis, lymphadenitis, skin disease and disseminated disease^3^.

NTM are typically environmental organisms that have been frequently isolated not only from water but also from soil, dust and plants^4,5^. NTM may occasionally infect humans and animals when they are exposed to contaminated environments. Although transmission of NTM between patients with cystic fibrosis has been suggested, the possibility of direct person-to-person transmission is small^6^. The highly significant difference in disease incidence among *Mycobacterium* species may be due to not only the genetic susceptibility of the host^7^ but also the different virulence potential among humans. Furthermore, different *Mycobacterium* species vary in host range as well as in capacity to infect human cells^8^.

*M*. *shigaense* has been isolated from skin specimens in three independent cases: a normal immunocompetent patient with a skin infection in China in 2013^9^, a patient with a history of Hodgkin’s disease and severe cellular immunodeficiency in 2013^10^, and in association with immune reconstitution syndrome in an acquired immune deficiency syndrome (AIDS) patient in 2016^11^. To date, only these three cases have been reported. In contrast to the well-studied *M*. *tuberculosis*, little is known about *M*. *shigaense*. To improve our understanding of the biology of *M*. *shigaense*, we used a systems biology approach to characterize its gene features and the host immune response.

In this study, we proposed that the new clinically isolated strain *M*. *shigaense* serves as a model of opportunistic mycobacterial pathogen rarely found in human disease. We compared the bacterial genome and transcriptome as well as the transcriptome of *M*. *shigaense*-infected host macrophages with those of *M*. *tuberculosis* and *M*. *leprae*, i.e., typical pathogenic bacilli, in order to further explore their capabilities to cause distinct diseases.

## Results

### Functional annotation of the completed genome of *M*. *shigaense*

**Fig. 1:**
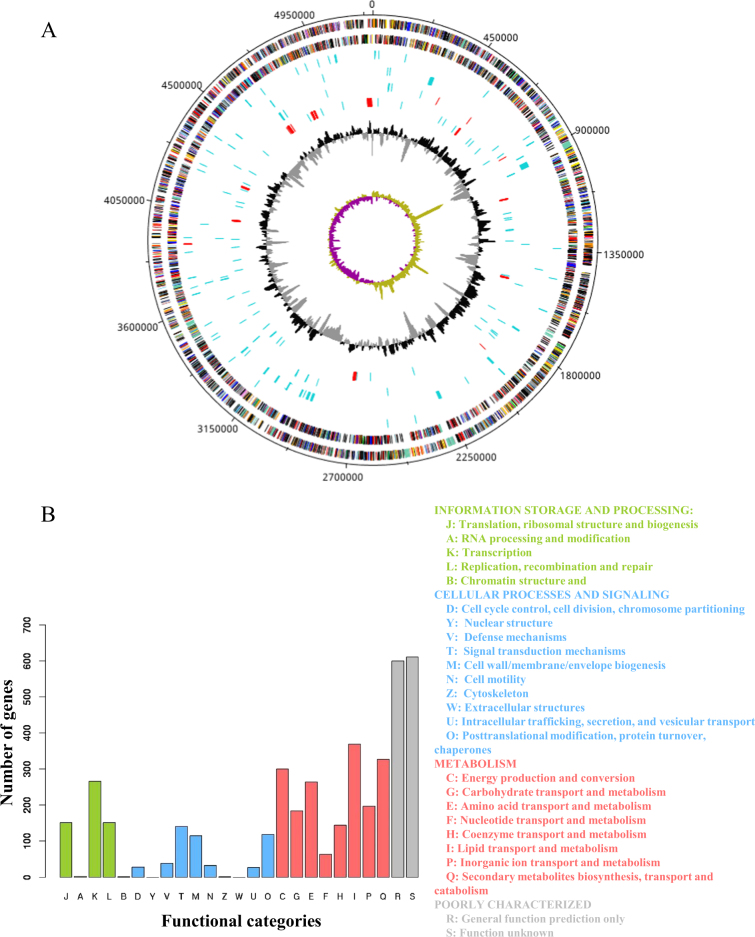
Overview of *M*. *shigaense* genome. **a** From outermost to innermost tracks are plus-strand protein-coding genes, minus-strand protein-coding genes, tRNAs (blue) or rRNAs (red, three rRNAs are too close to each other to be seen as separate genes at this scale), genomic islands, insertion sequences (blue) or CRISPRs (red), pseudogenes (blue) or prophage sequences (red), GC content and GC skew, respectively. **b** Functional categories of protein-coding genes by COG or NOG annotation. Four different colours and capital letter A to Z represent four top-level functional categories

The sequence data was generated with third-generation PacBio sequencing technology that generated 42,739 reads and second-generation Illumina Hiseq2000 sequencing technology that yielded 9,012,652 paired-end reads. The complete *M*. *shigaense* genome without any gaps (Supplementary Table S2) was assembled by taking advantage of PacBio long reads and the accuracy of Illumina Hiseq2000 short reads. The peak PacBio read length distribution was ~2 kb, and the maximum read length was over 12 kb (Supplementary Figure S1). The complete genome of *M*. *shigaense* was 5,207,883 bp in length, with 5098 predicted coding genes, 3 rRNAs and 47 tRNAs (Fig. 1a, Supplementary Tables S3, S4 and S5). The G+C content was estimated as 67.2%. These genome features of *M*. *shigaense* share certain similarities with the 12 other species used in the phylogenetic analysis of mycobacteria (Supplementary Table S6). A total of 48 pseudogenes were detected; this number was fewer than in the genomes of *M*. *avium*, *M*. *triplex* and *M*. *simiae*, which cluster with *M*. *shigaense*. Potential genomic features such as insertion of genomic islands, insertion sequences, clustered regularly interspaced short palindromic repeats (CRISPRs) and prophage sequences were also observed (Fig. 1a, Supplementary Table S7). In total, nearly 81% of 5,098 protein-coding genes could be functionally annotated by combining the cluster of orthologous groups (COG) and non-supervised of orthologous groups (NOG) databases. The functions of 1211 genes could not be predicted (categories R and S) (Fig. 1b).

### Comparative analysis of the *M*. *shigaense* genome

**Fig. 2:**
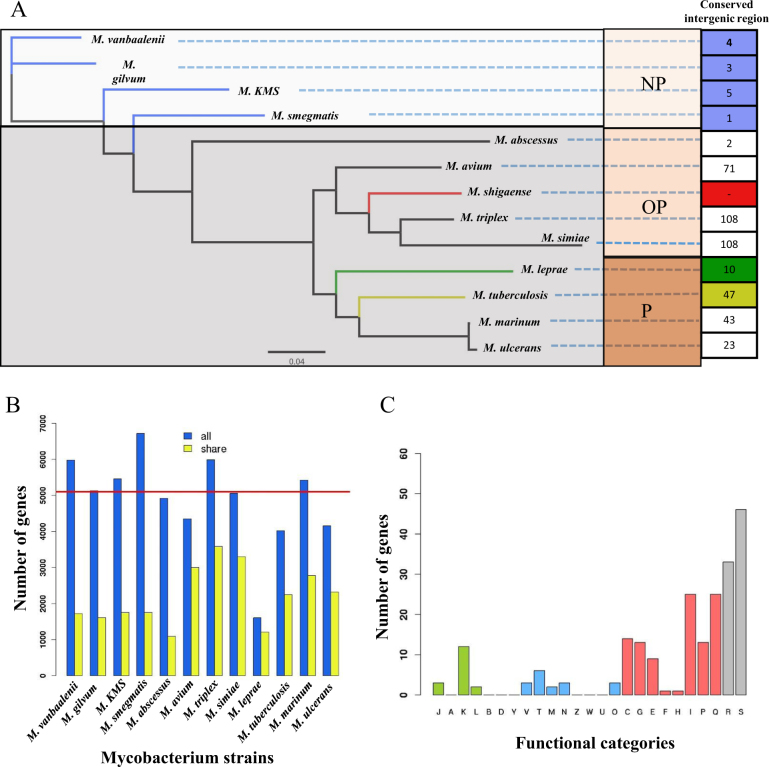
Phylogenetic analysis of the 13 selected Mycobacterium. **a** The phylogenetic tree built from 682 single-copy orthologous genes shared by all of 13 strains. Conserved intergenic region means the numbers of intergenic regions with high similarity between *M*. *shigaense* and each of the other 12 strains. **b** The numbers of homologous genes between *M*. *shigaense* and each of the other 12 strains. The red line indicates the number of *M*. *shigaense* genes. **c** Functional categories of genes with high similarity only for *M*. *shigaense*, *M*. *triplex*, *M*. *simiae* and *M*. *avium* (Fig. 1b shows meanings of different colours and capital letters). P pathogens, OP opportunistic pathogens, NP non-pathogens

The genome size, G + C content, and predicted protein-coding genes of *M*. *shigaense* are similar to those of other mycobacteria except *M*. *leprae*, which has a much smaller genome size (3.2 Mb), lower GC content (57.8%) and fewer protein-coding genes (*n* = 2,770) (Supplementary Table S6). On the basis of the orthologous gene analysis, 682 single-copy ortholog genes among 13 representative strains were obtained and used to construct a phylogenetic tree. All 13 strains separated into clades that aligned with two phenotypes, i.e., growth rate and pathogenicity. The phylogenetic tree was consistent with the hypothesis that *M*. *shigaense*, an opportunistic pathogen, would cluster with *M*. *avium*, *M*. *triplex* and *M*. *simiae*, which are all opportunistic pathogens (Fig. 2a). Moreover, the variation in the number of homologous genes of *M*. *shigaense* in each of the other 12 *Mycobacterium* species was highly consistent with their phylogenetic relationships (Fig. 2b). Although the 13 strains can be clustered into several evolutionary clades, *M*. *shigaense* and the other 12 strains indicated similar codon usage biases, which may be primarily due to the nucleotide composition of the genes (Supplementary Figure S2). The distribution peak of *M*. *shigaense* gene length is approximately 2 kb, with a maximum length of 23,025 bp and a minimum of 111 bp (Supplementary Figure S3).

We also performed a pairwise alignment of the *M*. *shigaense* whole genome with all 12 other strains. The basic idea is that if a gene from *M*. *shigaense* shows high similarity with *M*. *avium*, *M*. *triplex* and *M*. *simiae* but low similarity with the other nine strains, then it could be considered a specific gene in opportunistic pathogenic bacteria (opportunistic-specific gene). As a result, we identified 280 genes enriched in opportunistic species compared to *M*. *tuberculosis*, *M*. *leprae*, *M*. *ulcerans* and *M*. *marinum*, 236 of which were functionally annotated by the COG/NOG databases (Supplementary Table S4). Nearly half of the opportunistic species genes (42.79%) were involved in cellular metabolism, thereby indicating that these genes were possibly related to the adaptive metabolic process for the latency or activity of *M*. *shigaense* infection (Fig. 2c, Supplementary Figure S4). The rational phylogenetic clades validated that these 682 single-copy orthologous genes (Supplementary Table S8) are promising candidates for classifying new mycobacteria in the future.

### Potential virulence factors and antibiotic resistance of *M*. *shigaense*

Potential virulence genes of *M*. *shigaense* were predicted by aligning with curated virulence factors from bacterial pathogens from the commonly used virulence factors database VFDB^12^. However, no gene can be identified as a virulence gene because the highest identity was 47.6 (Supplementary Table S9). When compared directly with known *M*. *tuberculosis* virulence genes, two genes showed high similarity with esxN in the ESX-5 secretion system, whereas other homologs of the PE/PPE family of genes were not found. *M*. *shigaense* did not have mutations related to antibiotic resistance in the genes* rpoB*, *inhA*, *rpsL*, *rrs*, *embB*,* folP*, *gyrA* and *gyrB*, to rifampin, isoniazid, streptomycin, ethambutol, dapsone and moxifloxacin resistance, respectively. The genes *katG*, *erm*, *rrl*, and* pncA*, which are also associated with drugs treatment in *M*. *tuberculosis*, are absent in *M*. *shigaense*.

### Gene expression profile of *M*. *shigaense* cultured on L–J medium

**Fig. 3:**
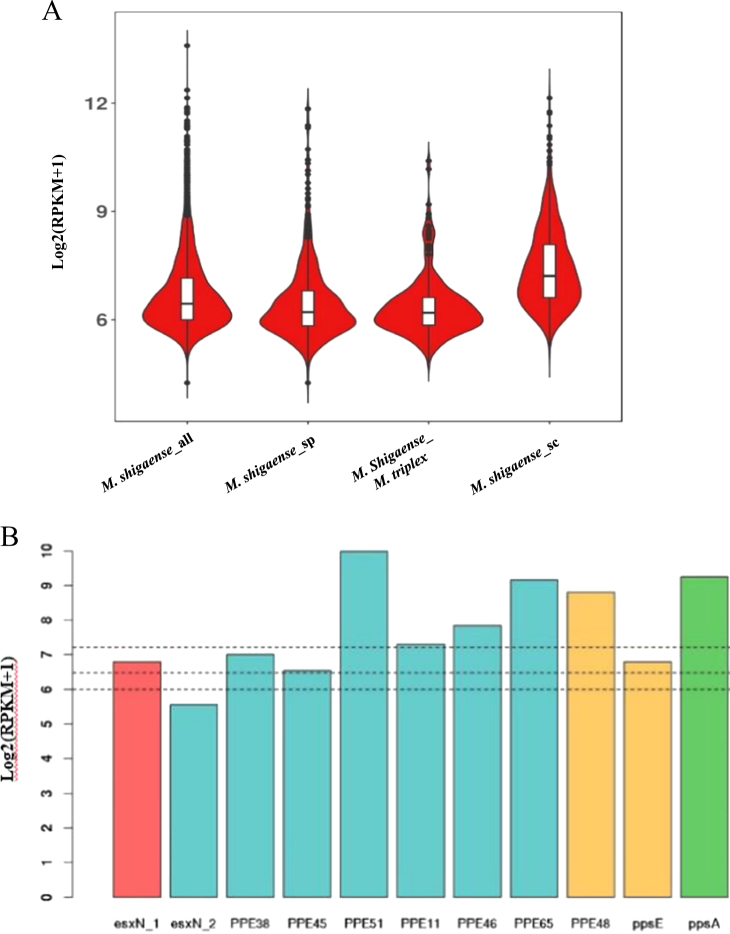
Comparisons of bacterial gene expression. **a** Distribution of expression levels of *M*. *shigaense* gene groups indicated by suffixes ‘all’ for all 5098 genes, ‘sp’ for specific (not in *M*. *triplex* or *M*. *tuberculosis*) and ‘sc’ for single-copy orthologous genes in three species. *M*. *shigaense_M*. *triplex* represent genes common to *M*. *shigaense* with *M*. *triplex* expressed in *M*. *shigaense*. **b** Gene expression of *M*. *shigaense* homologs of *M*. *tuberculosis* virulent genes. The three dashed lines represent the quartile of all gene expression levels in *M*. *shigaense*

To construct the expression profile of predicted protein-coding genes of *M*. *shigaense*, the transcriptome was sequenced under culture conditions. All the predicted genes of the *M*. *shigaense* genome were expressed, with maximum and minimum expression levels of 13.59 and 4.24 and quartiles of 6.00, 6.44 and 7.1, respectively, on the log2 scale (RPKM + 1) (Supplementary Table S4). The expression level of most genes was ~6 to 7, and few genes were expressed at a level of more than 10. We found no significant difference between plus- and minus-strand coded genes (Supplementary Figure S5). To further elucidate the relationship between predicted genes and transcripts, we performed de novo assembly of *M*. *shigaense* transcripts with a maximum length of 20,225 bp and a minimum length of 50 bp. Most of the transcripts (6665 of 8684) possessed lengths <1000 bp. Moreover, ~5000 transcripts were <500 bp, but only 40 of these could be mapped to *M*. *shigaense* protein-coding genes, thereby suggesting that the *M*. *shigaense* genome encodes many small RNAs that are approximately 50- to 500-nucleotide noncoding RNA molecules. Most of the transcripts (97.3%) assembled in the present study indicated unique genome positions (Supplementary Table S10). Notably, only 2086 of the 5098 protein-coding genes of *M*. *shigaense* were covered by 1103 de novo transcripts, with a maximum length of 20,225 bp and a minimum length of 182 bp. Some of these transcripts covered several continuous predicted genes on the same strand ((Fig. 3a, Supplementary Table S11).

The transcriptomes of two additional strains, *M*. *triplex* and *M*. *tuberculosis*, were obtained and sequenced (Supplementary Tables S12 and S13). Then, we examined the expression by *M*. *shigaense* of genes known to be associated with virulence of *M*. *tuberculosis*. All of the targeted genes of were expressed, with quartiles at 6.00, 6.45 and 7.2 on a log2 scale (RPKM + 1). As suspected, these virulence genes are indeed expressed by *M*. *shigaense* under culture conditions (Fig. 3b).

### Infection and replication capacities of *M*. *shigaense*, *M*. *triplex* and *M*. *tuberculosis* in macrophages

**Fig. 4:**
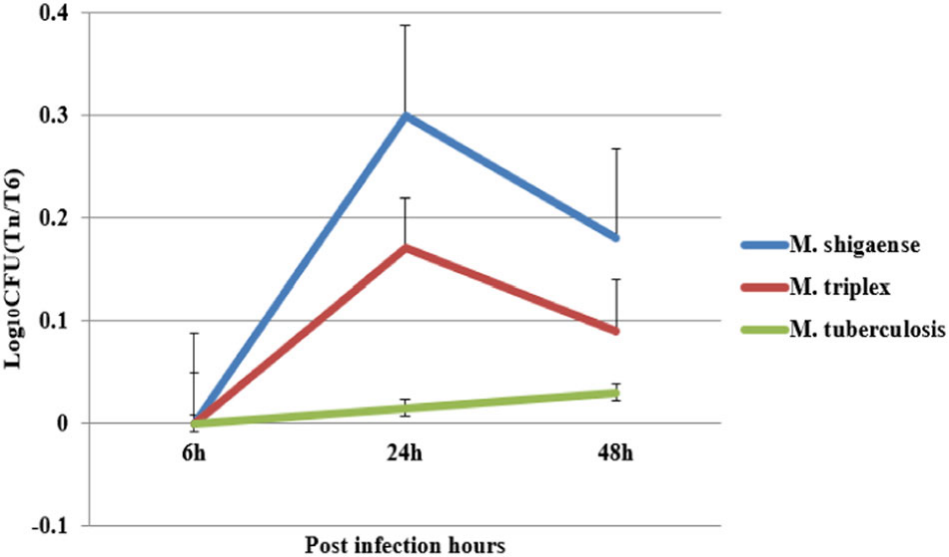
Intracellular viability of *M*. *shigaense*, *M*. *triplex*, and *M*. *tuberculosis*. Macrophages were infected with bacteria cultured on L–J medium. The intracellular bacteria were harvested from the macrophages at 6 h, 24 h, 48 h post infection and plated on L–J medium. The colony forming units were counted and plotted as Log_10_CFU counts. *M*. *shigaense* and *M*. *triplex* shared a similar trend of increase in Log_10_CFU from 6 h to 24 h post infection and a decrease from 24 h to 48 h post-infection, whereas *M*. *tuberculosis* underwent slow growth from 6 h to 48 h post-infection

To understand the different infection and replication capacities of different mycobacteria in macrophages, we used *M*. *shigaense*, *M*. *triplex* and *M*. *tuberculosis* to infect the human monocytic cell line THP-1 and compared the viability of the bacteria at three different time points. As shown in Fig. 4, similar replication patterns were observed for *M*. *shigaense* and *M*. *triplex* in THP-1 cells at 24 and 48 h, whereas the number of *M*. *tuberculosis* bacteria inside the macrophages showed no significant changes across time, thereby suggesting that *M*. *shigaense* and *M*. *triplex* can be inhibited by host innate immunity. However, these results did not apply to *M*. *tuberculosis*, which continued to grow slowly. NTM and *M*. *tuberculosis* had specific replication patterns.

### Common genes and pathways involved in the host response to different mycobacteria

**Fig. 5:**
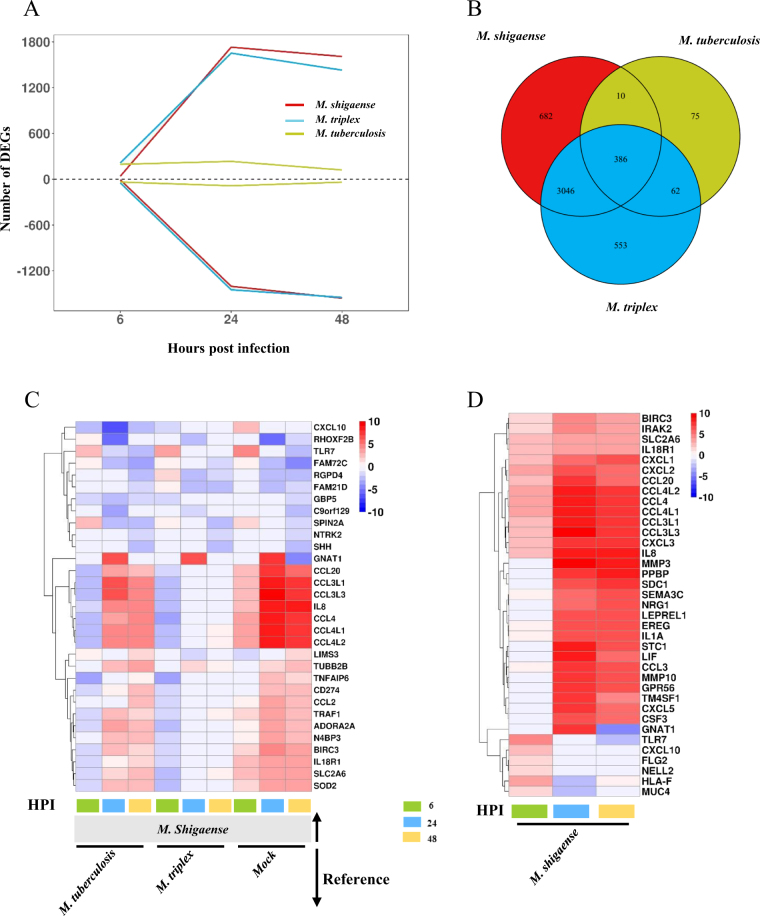
Overall gene expression signatures of post-infected macrophage by *M*. *shigaense*, *M*. *triplex* and *M*. *tuberculosis*. **a** The number of differentially expressed genes (DEGs) at three time points. Above zero indicates up-regulation. Below zero indicates down-regulation. **b** The number of overlapped DEGs between three strains. **c** DEGs only for *M*. *shigaense* compared with other two strains and mock group. HPI represents hours post infection. **d** Gene expression signature of top 20 upregulated DEGs from each time point of *M*. *shigaense*. Red indicates up-regulation, and blue indicates down-regulation. The colours (**c**, **d**) denote log2FC(Fold Change) of gene expression

Given that similar bacterial replication patterns may be associated with similar molecular patterns, host response transcriptomes were sequenced in the same samples used in the replication capacity experiments (Supplementary Table S14). Consistent with replication capacity in vitro, similar gene expression profiles were observed among macrophages infected with *M*. *shigaense* and *M*. *triplex* but not those infected with *M*. *tuberculosis* (Supplementary Figures S6A and B). Interestingly, the number of up- and downregulated differentially expressed genes (DEGs) in *M*. *shigaense-* and *M*. *triplex*-infected THP-1 cells increased significantly from 6 to 48 h, whereas DEGs in *M*. *tuberculosis-*infected cells only experienced a small fluctuation across post-infection times (Fig. 5a). However, among all infected macrophages, we still observed 386 common DEGs that are possibly essential in the initial host immune response (Fig. 5b). Moreover, we identified specific DEGs expressed in *M*. *shigaense*-infected cells compared with *M*. *triplex*- and *M*. *tuberculosis*-infected cells and the mock group (Fig. 5c). In addition, the top 20 upregulated genes for *M*. *shigaense* at each time point were not completely the same; however, most chemokines were expressed at relatively high levels in *M*. *shigaense* group (Fig. 5d).

To further understand the biological functions of the DEGs among the three mycobacterial strain-infected macrophages, we performed KEGG pathway enrichment analysis to determine the cellular pathways with significant perturbation. As shown in Supplementary Figure S7, Toll-like and NOD-like receptors were more significantly disturbed than RIG-I-like receptors, which mainly recognize double-strand RNAs of the virus. Despite the low-level expression of the DEGs, the perturbed innate immunity-related pathways (NF-κB signaling pathway, TNF signaling pathway, cytokine-cytokine interaction pathway and chemokine signaling pathway) induced by *M*. *tuberculosis w*ere similar to those induced by *M*. *shigaense* and *M*. *triplex*. These results indicate that the host cell responses for *M*. *tuberculosis* and other two strains shared common early-response and late-response pathways (Supplementary Figure S7). Nevertheless, *M*. *tuberculosis* seemed to cause the most significant perturbation for the cytokine-cytokine interaction pathway in host cells. Interestingly, we found two pathways that were not significantly perturbed by *M*. *tuberculosis* in THP-1 cells, i.e., cell cycle and DNA replication pathways, in which genes are significantly downregulated for *M*. *shigaense* and *M*. *triplex*. Thus, host response differentiations supported the finding that *M*. *tuberculosis* can escape host immune surveillance.

### Specific genes involved in the host response to different mycobacteria

As shown above, thousands of host genes were significantly differentially expressed in response to different mycobacteria. To reveal the potential genes related to the specific host response to three mycobacterial strains, the transcriptomes of infected THP-1 macrophages were compared. First, we highlighted 11 DEGs from *M*. *shigaense*- and *M*. *triplex*- infected vs. *M*. *tuberculosis*- infected cells, which were selected as specific genes of opportunistic pathogenic bacteria compared with pathogenic bacteria (Figure 5c).* CCL4* was much more upregulated in *M*. *shigaense-* and *M*. *triplex*-infected cells than in the mock group and *M*. *tuberculosis-*infected cells. Among *M*. *shigaense*-specific DEGs (Fig. 5d), two genes, namely, *TLR7* and *GNAT1*, were consistently upregulated at 6 or 24 h in *M*. *shigaense*-infected macrophages, respectively. In *M*. *tuberculosis*-infected macrophages (Supplementary Figure S6), some well-known antivirus genes involved in the innate immune response as part of the host defense response to clear viral infections were specifically upregulated, including *CMPK2*, *OAS2*, *SIGLEC1*, *IFITM1*, *IFIT1* and *RSAD2*, and in particular *IFI44L* and *TRIM22*. These genes play an important role against HIV-1 infection in the macrophage^13^.

**Fig. 6:**
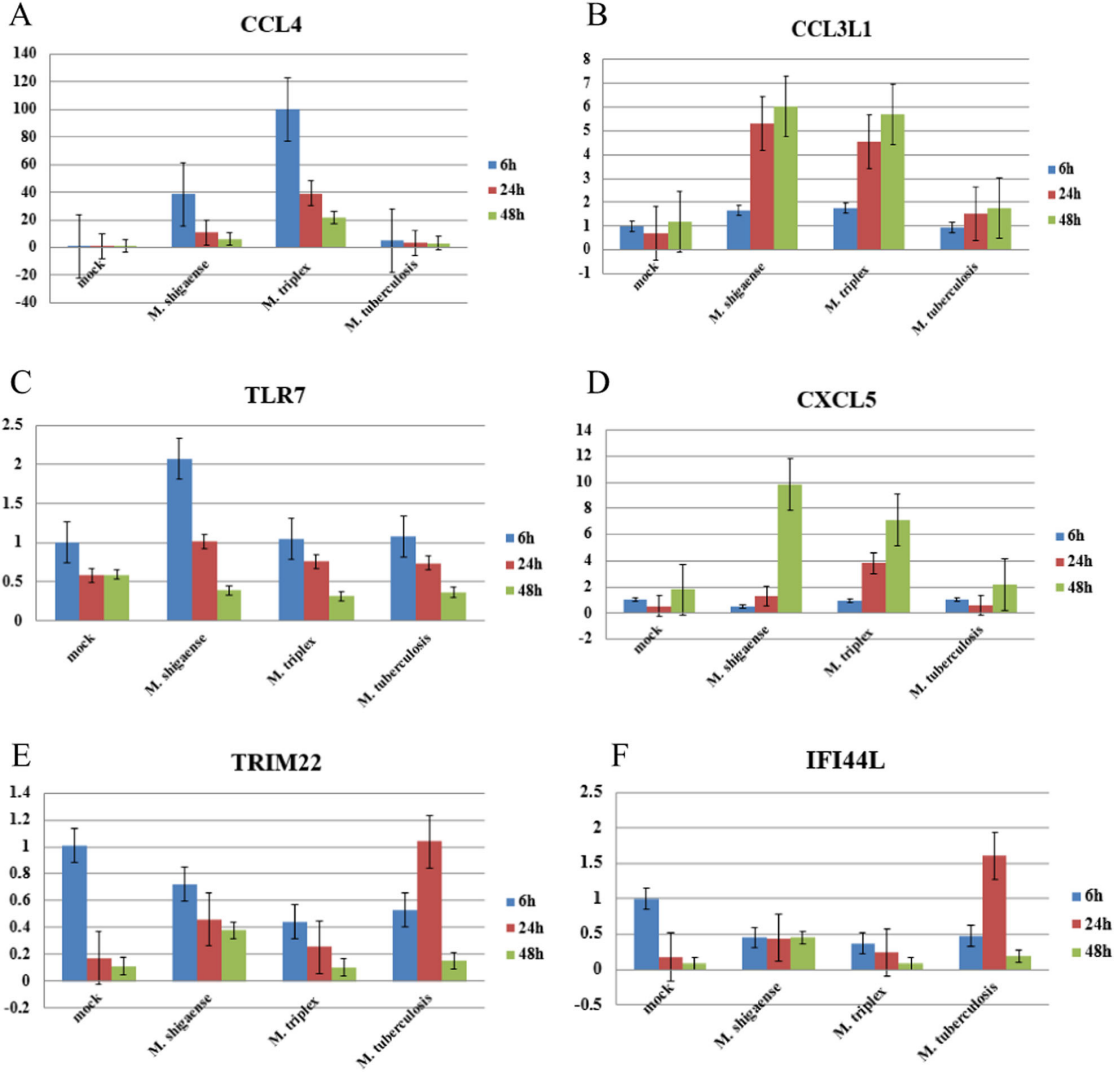
THP-1 derived macrophage special genes quantified using RT-PCR. Six differentially expressed genes (**a**–**f**) were detected at 6, 24 and 48 h post infection among *M*. *shigaense*, *M*. *triplex*, *M*. *tuberculosis* and mock group. Data are expressed as the mean and standard deviation (±) of three independent experiments. The expressed levels of special genes were in accordance with the results of RNA sequencing

Based on RNA sequencing data, six genes, including *CCL4*, *CCL3L1*, *CXCL5*,* TLR7*, *TRIM22* and *IFI44L*, were selected to validate the accuracies of the gene expression data from RNA-Seq via RT-PCR (Fig. 6). Quantifications by RT-PCR of transcript levels were consistent with RNA-Seq, except for *CCL3L1*, which is possibly a candidate NTM-specific gene missed by high-throughput selection.

## Discussion

Despite extensive genome and transcriptome research on many *Mycobacterium* species, systematic studies on opportunistic pathogens are rare. In this study, we report the first whole-genome sequencing and analysis of *M*. *shigaense*, a new opportunistic pathogen that was isolated from a patient with a cutaneous infection. We also investigated the commonalities and differences in the profiles of virulence genes in the genome, host cell response genes and perturbed pathways during infection by opportunistic pathogenic vs. pathogenic bacteria.

*M*. *shigaense*, with a genome size of 5.21 Mbp, was similar to the opportunistic pathogens *M*. *avium* (4.83 Mbp), *M*. *simiae* (5.94 Mbp) and *M*. *triplex* (6.38 Mbp) but was larger than pathogenic bacteria, e.g., *M*. *tuberculosis* (4.41 Mbp) and *M*. *leprae* (3.27 Mbp), which have smaller genomes. *M*. *shigaense* belongs to the clade of opportunistic pathogens, which have more homologous genes than pathogenic bacilli. Via a phylogenetic tree built from a set of 682 orthologous proteins in 13 representative species, we found that pathogenic and non-pathogenic species were naturally divided, thereby forming separate clusters. In addition to these two clusters, an independent cluster contained several opportunistic pathogenic bacteria in accordance with clinical features. Intriguingly, *M*. *marinum* and *M*. *ulcerans* resided together in a cluster that consists of slowly growing and pathogenic bacteria, thereby providing genetic support that *M*. *marinum* and *M*. *ulcerans* should be classified as pathogens rather than as opportunistic pathogens. This observation fits with the recognition that *M*. *ulcerans* is responsible for Buruli ulcers, the third most common mycobacterial infection in the world after tuberculosis and leprosy. Cases of Buruli ulcers have been reported from at least 32 countries. The condition usually occurs in communities adjacent to rivers, swamps and wetlands^14^. Feng et al. described an outbreak of cutaneous *M*. *marinum* infection in China in 2008^15^. Thus, *M*. *marinum* and *M*. *ulcerans* were categorized into the pathogenic group based on gene analysis and clinical practice. An *in silico* screen for known mycobacterial virulence genes indicated that *M*. *shigaense* underwent deletions in the genes for lipid biosynthesis, including phthiocerol dimycocerosates and phenolic glycolipids. Homologs of *ppsA* and *ppsE* *M*. *tuberculosis* genes, but not the full complement of PDIM or PGL biosynthetic genes, can be found in *M*. *shigaense*.

The genes and secreted proteins ESAT-6 and CFP-10^16^, encoded by ESX gene systems, were absent in *M*. *shigaense*. ESX mutants of *M*. *tuberculosis* have been previously demonstrated to be avirulent, failing to lyse infected macrophages, thereby implicating the involvement of secreted ESAT-6 in the mediation of virulence through host cell cytolysis^17^. A third set of genes implicated in *M*. *tuberculosis* virulence factors are the large PE/PPE family. Only seven PPE genes were expressed in *M*. *shigaense*. The inability to produce these lipids and secreted compounds, which are used by *M*. *tuberculosis* during its adaptation to the host, may be associated with the lower virulence and opportunistic pathogenicity of *M*. *shigaense*.

As a practical matter, the presence or absence of virulence-associated genes in the *M*. *shigaense* genome could be used to design molecular diagnostic tools. Moreover, the genes *katG*, *erm*, *rrl*, and *pncA*, which encode targets of the drugs isoniazid, clarithromycin and pyrazinamide in *M*. *tuberculosis*, are absent in *M*. *shigaense*. Clinically, the patient, from which *M*. *shigaense* was isolated, obtained a good treatment effect using moxifloxacin, which targets Gyrase A encoded by the genome, thereby supporting our predictions of drug susceptibility (Supplementary Table S15).

Another interesting observation is that *M*. *shigaense* only possesses 48 pseudogenes, which is fewer than all other strains except *M*. *tuberculosis*. Though pseudogenes have mutations compared to their paralogous genes and are considered inactive, there are reports that their transcripts can have a role in gene regulation in many biological processes^18^. The limited number of pseudogenes in the genome of *M*. *shigaense* indicates that it did not undergo the scale of reductive evolution described for *M*. *leprae* and *M*. *ulcerans*. Host and or tissue specificity are attributed to such downsizing^19^.

A comparison of *M*. *shigaense* with the *M*. *tuberculosis* and *M*. *triplex* transcriptomes showed that while most of the transcripts (97.3%) corresponded to unique genome positions, some of the transcript covered several continuous predicted genes on the same strand (Supplementary Tables S12, and S13). The reason for this result is gene conservation in *Mycobacterium* species, which indicates an ancient, close history and then likely a principal role in mycobacterial physiology. Earlier studies^20,21^ have reported that many of these genes were obtained via horizontal gene transfer for the generation of divergence among the currently studied *Mycobacterium* species, and this result was consistent with our findings.

Moreover, *M*. *shigaense* had different infection and replication capacities compared with *M*. *tuberculosis* in infected macrophages but similar capacities to those of the opportunistic pathogen *M*. *triplex*. Other studies showed similar results. Martinez et al. found that the number of *M*. *fortuitum* that remained inside macrophages increased by two-fold from 6 to 48 h post-infection, whereas *M*. *tuberculosis* persisted inside macrophages without causing cell damage and without inducing reactive oxygen species because it grows slowly during the first 48 h^22^. *Mycobacterium species* differ in replication capacity in human cells, thereby suggesting that species-specific mechanisms might be involved in mycobacterial infection and replication in human cells.

Common and specific host gene expression in response to infection with three species were compared. Host cell transcriptome patterns were generally consistent with previous findings. This study is limited in the number of replicates of RNAseq libraries studying the transcriptomes of bacteria grown in axenic media and of infected macrophages due to the high costs of RNAseq. Thus, we used GFOLD to compensate for this lack of replicates. We also conducted three independent macrophage infection experiments. The RNAseq and RT-PCR data between trials showed low variability, with the exception of *CCL3L1*. *CCL4* modulated immune responses to mycobacterial infection by increasing phagocytosis and suppressing the growth of *M*. *tuberculosis* within macrophages^23,24^. Another interesting gene is *CXCL5*, which was found to be downregulated in *M*. *tuberculosis-*infected macrophages. Nouailles et al^25^. presented a detailed analysis and showed that *CXCL5* was mainly involved in pulmonary PMN attraction. *TLR7* was consistently upregulated in *M*. *shigaense* infection. TLRs are the first line of defense against *mycobacteria*^26^ and may have a role in decreasing the viability of *M*. *shigaense* in infected macrophages^27,28^. *IFI44L* is related to interferon-mediated inflammation in tuberculosis infection, such as lymph node tuberculosis and viral diseases^29^. *TRIM22* is constitutively expressed at high levels in monocytes and plays important roles in inflammation^30^.

In this study, we exploited *M*. *shigaense* as a unique resource to conduct the most comprehensive study of the differential gene features in *Mycobacterium species*. We provided insights into the genome characteristics of *M*. *shigaense* and the mechanisms of host-pathogen interactions using comprehensive comparative analysis of the bacterial genome and the host response transcriptome. These data will help to elucidate how increased variability of gene expression and effective adaptability potentially affect the pathogenicity of mycobacteria and the immune processes of the host.

## Materials and methods

### Ethics statement

Written informed consent was obtained from all patients, and all samples were anonymized in the investigations that led to the isolation of *M*. *shigaense*^9^. The study was reviewed and approved by the institutional ethics review board of the Institute of Dermatology, Chinese Academy of Medical Sciences and Peking Union Medical College, Nanjing, Jiangsu, China (No. 2012-KY-021).

### Bacterial strains and genome information

*M*. *shigaense* used in this study was isolated from a chronic cutaneous mycobacterial infection in an immunocompromised woman at the Institute of Dermatology, Jiangsu, China^9^. *M*. *triplex* and *M*. *tuberculosis* were purchased from ATCC. They were cultured on Middlebrook 7H10 agar with Middlebrook OADC enrichment at 37 °C.

Genome information from 12 *Mycobacterium* species was downloaded from the NCBI genome database. The genome annotation versions are as follows: *M*. *leprae* (*ASM19585v1*), *M*. *tuberculosis* (*ASM19595v2*), *M*. *marinum* (*ASM1834v1*), *M*. *ulcerans* (*ASM1392v1*), *M*. *avium* subspecies (*ASM786v1*), *M*. *triplex* (*BN973_1*), *M*. *simiae* (*ASM158476v1*), *M*. *abscessus* subspecies (*ASM6918v1*), *M*. *vanbaalenii* (*ASM1530v1*), *M*. *gilvum* (*ASM18443v1*), *M*. *KMS* (*ASM1540v1*), and *M*. *smegmatis* (*ASM1500v1*).

### Genome sequencing and de novo assembly of *M*. *shigaense*

Total *M*. *shigaense* genomic DNA was extracted from cultured cells using a QIAamp DNA mini kit (Qiagen, Venlo, The Netherlands) according to the manufacturer’s instructions. The purity and quantity were assessed by measuring the absorbance at 260 nm and 280 nm using a NanoDrop 2000 spectrophotometer. Gel electrophoresis was carried out in 1.0% agarose gel at 100 mV for 45 min for visualization and confirmation of the quality of high-molecular-weight genomic DNA. The raw genome sequence was generated using the Illumina Hiseq2000 system, and the raw sequences were preprocessed with Trimmomatic (version 0.35)^31^. Trimming of raw reads was performed as follows: bases with quality of less than 10 and drop reads less than 36 were eliminated.

The complete assembly of the *M*. *shigaense* genome was conducted by standard procedures described previously^32^. The accession number of the genome that we registered at NCBI is SUB2955739. Briefly, reads generated from the Illumina Hiseq2000 were first extended to long sequence fragments using Velvet (version 1.2.10)^33^. Then, sequencing errors in the PacBio long reads were corrected by aligning with the short-read sequences from the Hiseq2000 using BLASR (version 3.1)^34^. Third, the draft genome was generated using a Celera assembler (version 8.3)^35^. Finally, SOAP GapCloser (version 1.10) was used to close gaps in the draft genome^36^. The protein-coding genes, rRNAs and tRNAs of *M*. *shigaense* were predicted using NCBI glimmer server (version 3.0)^37^, RNAmmer server (version 1.2)^38^ and tRNAscan-SE server (version 2.0)^39^, respectively.

The functions of protein-coding genes were annotated using the NCBI nr and eggNOG databases (version 4.0)^40^. Genomic islands, insertion sequences, CRISPR sites and prophage sequences were predicted using Island Viewer server^41^, ISsaga server (http://issaga.biotoul.fr/issaga_home.php), CRISPR Finder server^42^ and PHAST server^43^, respectively. The potential pseudogenes of *M*. *shigaense* were observed by pairwise alignment between protein-coding genes and known pseudogenes of the other 12 genomes, with identity ≥ 80 and subject sequence coverage ≥ 60. Functional annotations of the *M*. *shigaense* genome were drawn in tracks on the circular genome maps using DNAPlotter^44^.

### Comparative genome analysis of *M*. *shigaense* genome

The phylogenetic tree was built as follows: identification of a group of orthologous proteins among 13 Mycobacterium species using OrthoMCL (version 2.0.9)^45^, multiple sequence alignments of the ortholog group using MAFFT (version 7.025)^46^, and concatenation of multiple sequence alignments and estimation of maximum likelihood using PyML (version 3.1)^47^ with 100 bootstraps. Gene order conservation analysis was conducted using DAGchainer^48^. The pairwise alignments of the whole genome were performed using LASTZ (version 1.03.54) and UCSC tools, including axtChain, chainNet and netToAxt. Relative synonymous codon usage (RSCU) scores were calculated with CodonW (version 1.3) to evaluate the codon usage bias of each species. All pairwise alignments were conducted using NCBI BLAST software (version 2.4.0 + ).

### Assays for viability of mycobacteria after infection of THP-1 cells

The human monocytic cell line THP-1 (ATCC TIB 202) was cultured in RPMI 1640 medium supplemented with 10% fetal bovine serum (Gibco, Invitrogen, Saint Aubin, France) at 37 °C with 5% CO_2_. The cells were transferred to a 24-well plate at 1.5 × 10^5^ cells per well and pretreated with 100 µg/mL phorbol 12-myristate 13-acetate for 48 h to induce differentiation into macrophages. Mycobacteria (*M*. *shigaense*, *M*. *triplex* and *M*. *tuberculosis*) were added to the macrophage culture in triplicate wells at a multiplicity of infection (MOI) of 1 in order to generate a detectable immune response^22^. A mock infection was performed with culture medium. After 6 h at 37 °C and 5% CO_2_, the infected macrophages were washed with 1 × Hanks solution (Gibco) to remove extracellular mycobacteria and then incubated with fresh medium. At 6, 24, and 48 h, the cells were lysed with 0.05% Tween-20 (Sigma Aldrich) and plated on Löwenstein–Jensen (L–J) medium. Colony forming units (CFUs) from the infected cells were compared with those of the original inoculum size of mycobacteria plated directly on L–J medium. The entire experiment was repeated thrice, thereby yielding a total of nine wells that included three wells per species, including mock-infected cells.

### RNA isolation, QC and sequencing

Each of the three mycobacterial species were cultured in Middlebrook 7H9 medium with Middlebrook OADC enrichment at 37 °C and then pelleted by centrifugation. The cells were lysed and processed to obtain total RNA using reagents provided in the QIAamp RNA mini kit according to manufacturer’s instructions (Qiagen, Venlo, The Netherlands). For obtaining RNA from infected macrophages, triplicate wells of THP-1 cells were infected with mycobacteria and harvested at three time points, including 6, 24, and 48 h, as described in the previous section. The washed cells from a total of 9 wells for each group were pooled from three independent experiments. The washed cells were lysed using the reagents in the QIAamp RNA mini kit. RNA quantity and quality were estimated with a Qubit 2.0 Fluorometer and Agilent Technology 2100 Bioanalyzer, respectively. RNA libraries were assessed using a Qubit and an Agilent 2100 Bioanalyzer, and differentially expressed genes were analyzed via qPCR. The samples were then subjected to sequencing on an Illumina HiSeq platform.

### Analysis of bacterial and host transcriptomes

Reference-based mapping of bacterial transcriptomes and de novo-assembled transcripts of *M*. *shigaense* were predicted using Rockhopper (version 2.03)^49^. Host transcriptomes were aligned with HISAT2^50^, and differentially expressed genes (DEGs) were identified using GFOLD (version 1.1.4)^51^, which indicated improved performance in the absence of replicated samples. KEGG pathway enrichment analysis was based on Fisher’s exact test with 22,810 human protein-coding genes as background.

### Quantitative real-time PCR (qPCR) assay

The expression levels of the genes *CCL4*, *GNAT1*, *CCL3L1*, *TLR7*, *DSCAM* and* PPBP* were measured by qPCR using an ABI 7300 qPCR instrument (primer details are listed in Supplementary Table S1). Fold differences in expression levels among the RNA samples were verified by repeating the THP-1 infection experiment, extracting the RNA and performing qPCR for the panel of genes as described previously, which were calculated via 2^−^^ΔΔCT^ method after normalization to β-actin.

### Data analysis

Statistical significance was determined with GraphPad Prism 5 software. ANOVA was used for comparisons involving three or more groups. All values are expressed as the mean ± SEM, and *p* < 0.05 is considered significant.

## Electronic supplementary material

Supplementary Figure S1

Supplementary Figure S2

Supplementary Figure S3

Supplementary Figure S4

Supplementary Figure S5

Supplementary Figure S6

Supplementary Figure S7

Supplementary Table S1

Supplementary Table S2

Supplementary Table S3

Supplementary Table S4

Supplementary Table S5

Supplementary Table S6

Supplementary Table S7

Supplementary Table S8

Supplementary Table S9

Supplementary Table S10

Supplementary Table S11

Supplementary Table S12

Supplementary Table S13

Supplementary Table S14

Supplementary Table S15
