# Supplementary figures and images for: Landscape of the genome and host cell response of Mycobacterium shigaense reveals pathogenic features
Source: Emerg Microbes Infect. 2018 Jun 22;7:112. doi: 10.1038/s41426-018-0116-z (PMC6015043; doi:10.1038/s41426-018-0116-z)

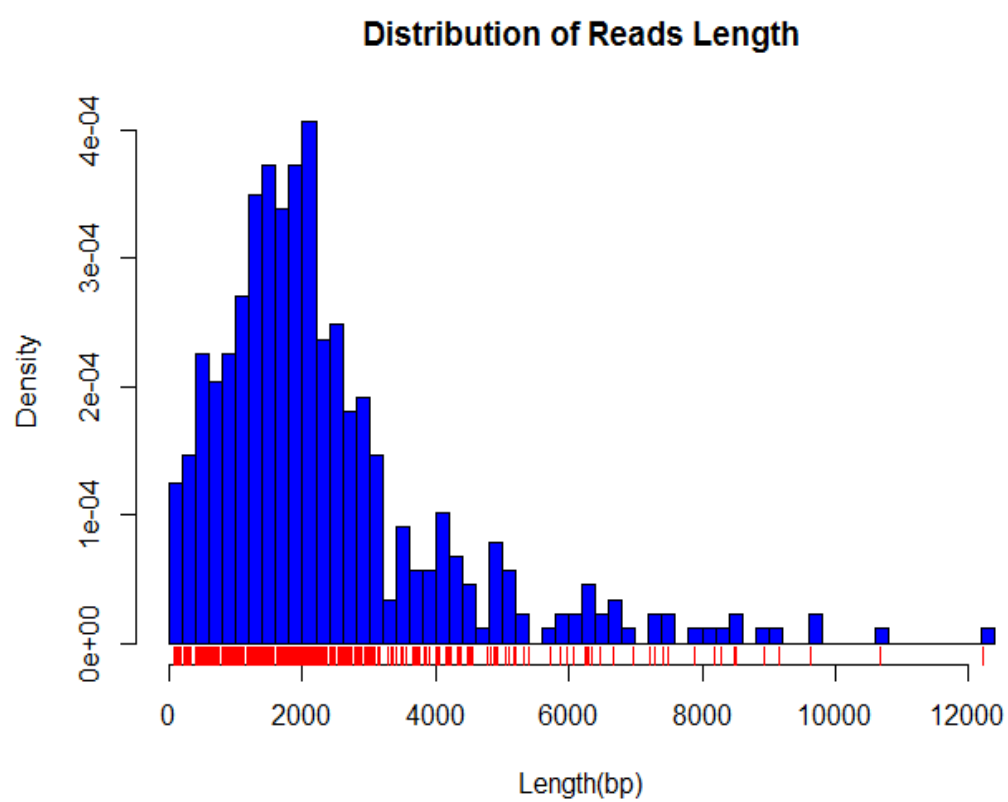

**Supplementary Figure S1.** Length distribution of PacBio raw reads

Supplement: Supplementary file 1 — Supplementary Figure S1 [file 41426_2018_116_MOESM1_ESM.pdf]
