# Supplementary material for: Landscape of the genome and host cell response of Mycobacterium shigaense reveals pathogenic features
Source: Emerg Microbes Infect. 2018 Jun 22;7:112. doi: 10.1038/s41426-018-0116-z (PMC6015043; doi:10.1038/s41426-018-0116-z)
Supplement: Supplementary file 2 — Supplementary Figure S2 [file 41426_2018_116_MOESM2_ESM.pdf]

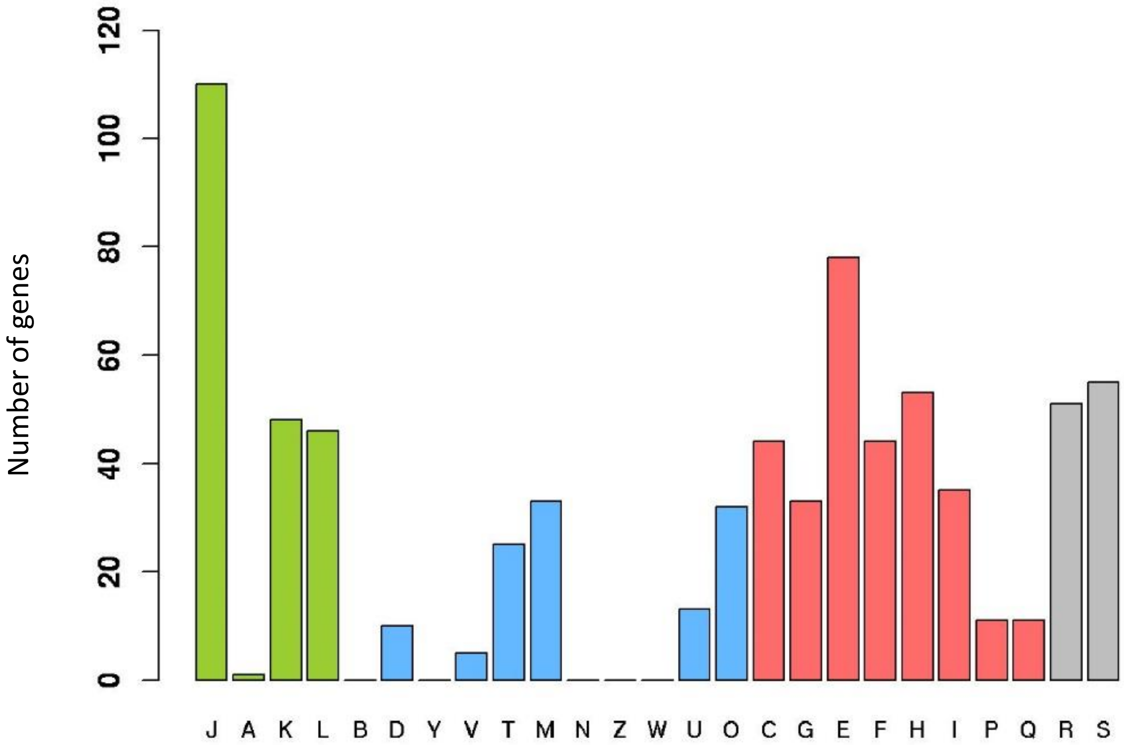

Supplementary Figure S2. Functional categories of single copy ortholog genes used to build the phylogenetic tree (See Figure 1B for meanings of different colors and capital letter and Supplementary Table S8).
