# Supplementary material for: Landscape of the genome and host cell response of Mycobacterium shigaense reveals pathogenic features
Source: Emerg Microbes Infect. 2018 Jun 22;7:112. doi: 10.1038/s41426-018-0116-z (PMC6015043; doi:10.1038/s41426-018-0116-z)
Supplement: Supplementary file 3 — Supplementary Figure S3 [file 41426_2018_116_MOESM3_ESM.pdf]

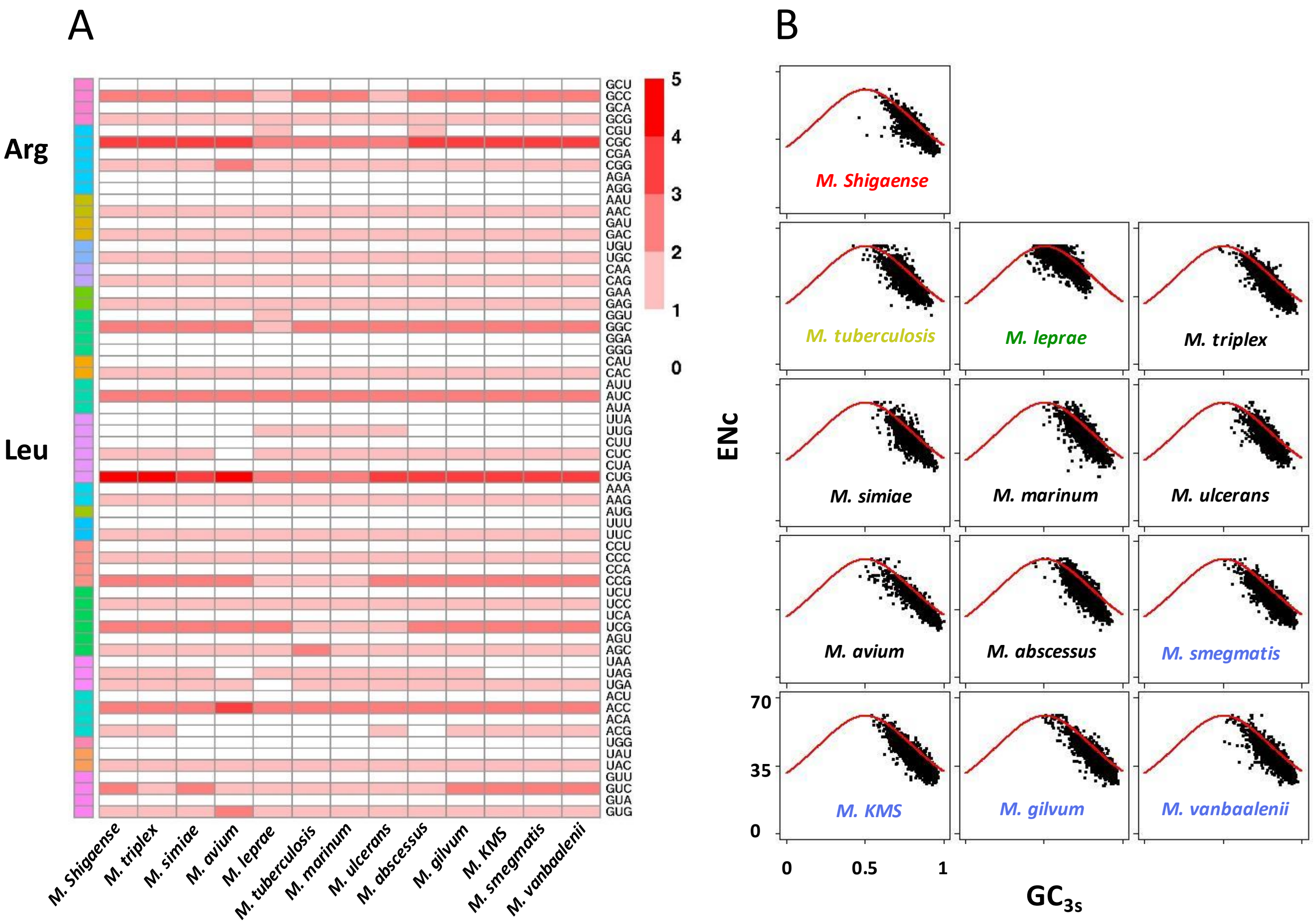

Supplementary Figure S3. Analysis of codon usage bias (CUB) for the 13 strains. (A) The RSCU analysis of preferred codons for each of 20 amino acids. (B) The ENc-plot. The standard curve (red color) ENc = $2+GC_{3s}+29/[GC_{3s}^2+(1-GC_{3s})^2]$ represents the expected ENc to GC_{3s}. ENc represents the effective number of codons, and GC_{3s} for the third synonymous codon position GC content. If genes follow the standard curve, CUB is determined primarily by the nucleotide composition of genes.
