# Supplementary material for: Landscape of the genome and host cell response of Mycobacterium shigaense reveals pathogenic features
Source: Emerg Microbes Infect. 2018 Jun 22;7:112. doi: 10.1038/s41426-018-0116-z (PMC6015043; doi:10.1038/s41426-018-0116-z)
Supplement: Supplementary file 4 — Supplementary Figure S4 [file 41426_2018_116_MOESM4_ESM.pdf]

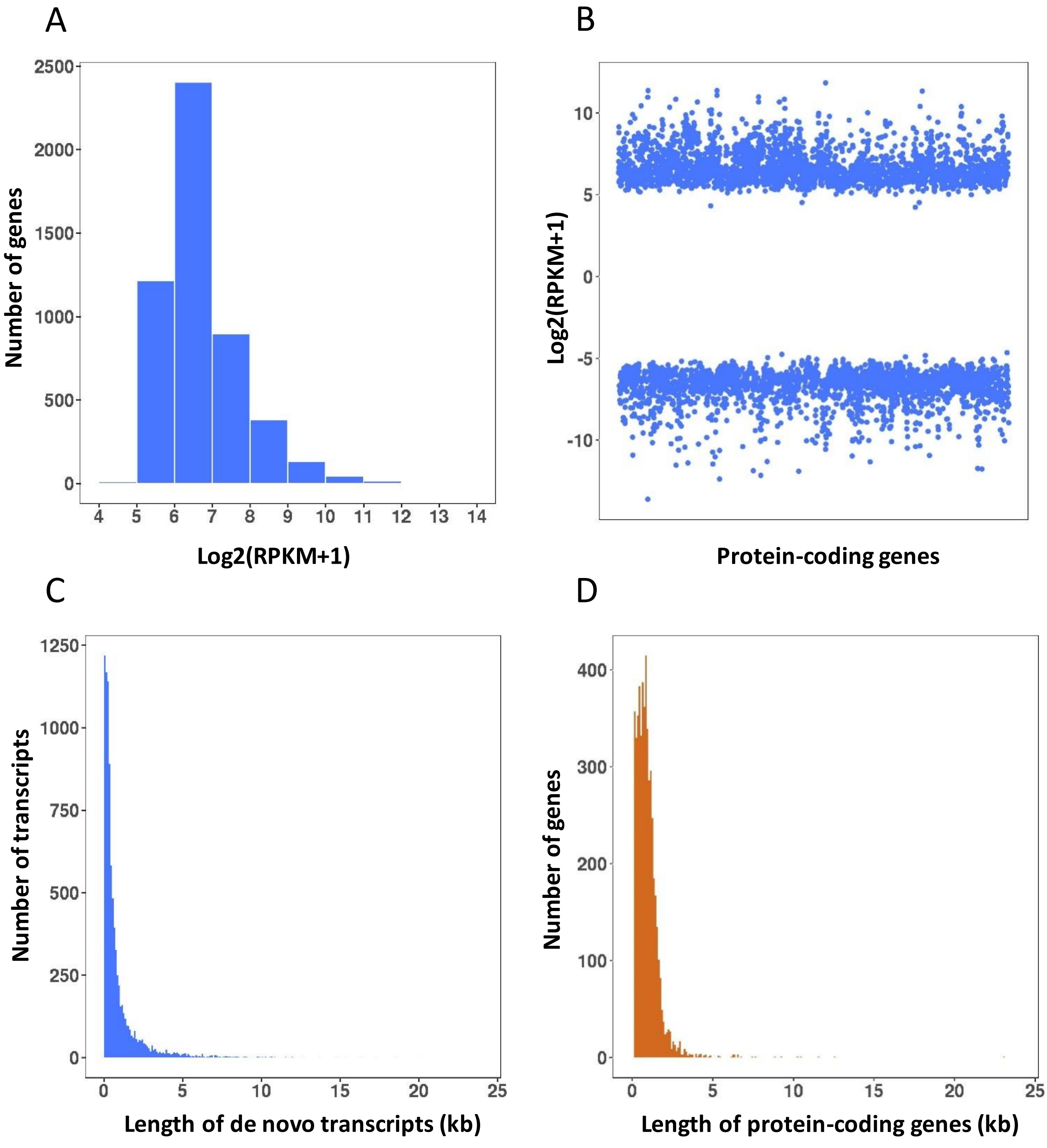

Supplementary Figure S4. Informations of *M. shigaense* protein-coding genes. (A) Expression distribution of *M. shigaense* protein-coding genes. (B) Expression values of *M. shigaense* protein-coding genes. Plus values are for plus-strand genes, and minus values for minus-strand genes. (C,D) Length distribution of de novo transcripts and protein-coding genes of *M. shigaense*.
