# Supplementary material for: Landscape of the genome and host cell response of Mycobacterium shigaense reveals pathogenic features
Source: Emerg Microbes Infect. 2018 Jun 22;7:112. doi: 10.1038/s41426-018-0116-z (PMC6015043; doi:10.1038/s41426-018-0116-z)
Supplement: Supplementary file 5 — Supplementary Figure S5 [file 41426_2018_116_MOESM5_ESM.pdf]

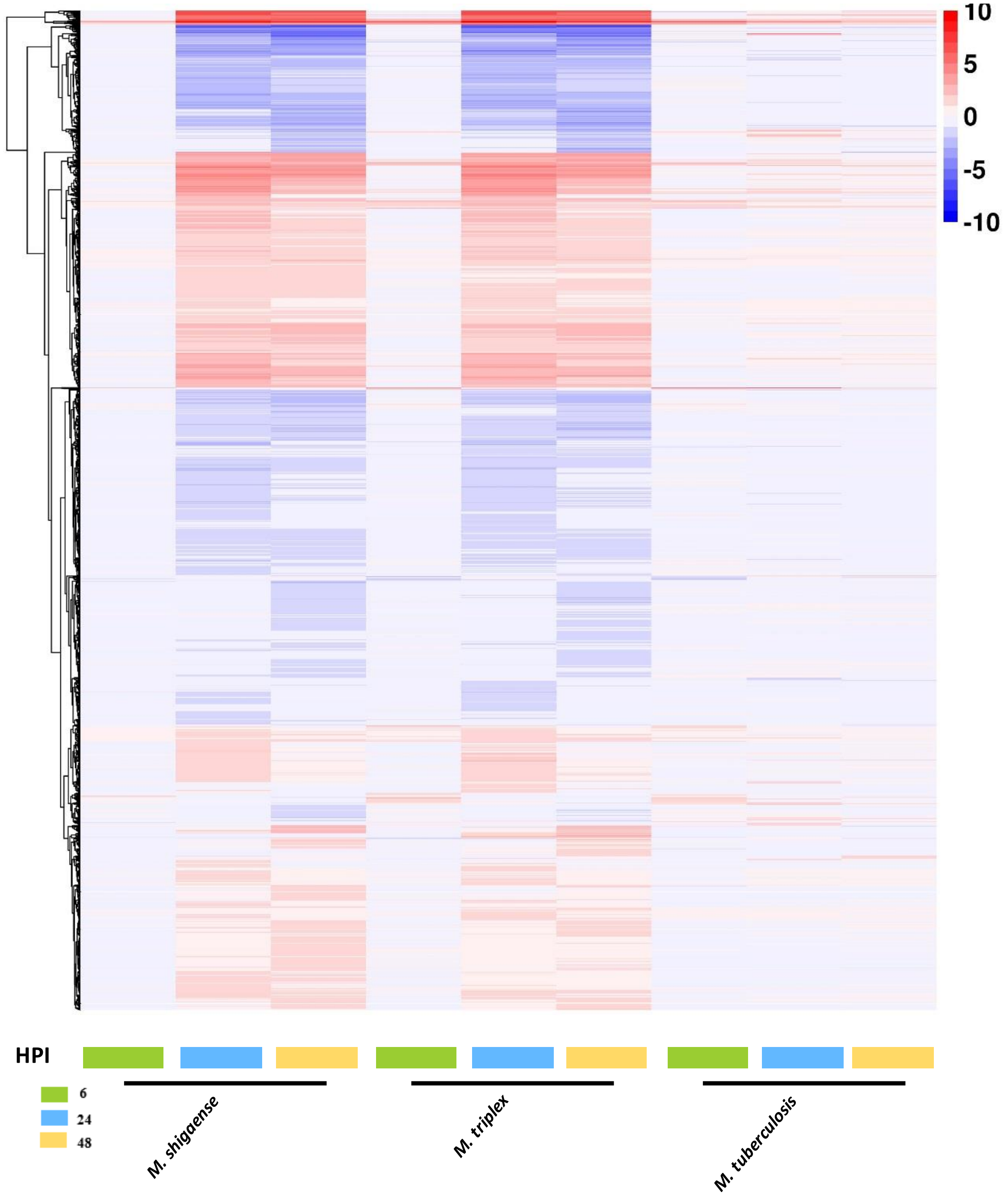

Supplementary Figure S5. Gene expression signature of all of DEGs (Absolute value of log2FC >= 1 and FDR <= 0.01) from each time point for *M. shigaense*, *M. triplex* and *M. tuberculosis*. The colors denote log2FC of genes expression. HPI has the same meanings with Figure 5.
