# Supplementary material for: Landscape of the genome and host cell response of Mycobacterium shigaense reveals pathogenic features
Source: Emerg Microbes Infect. 2018 Jun 22;7:112. doi: 10.1038/s41426-018-0116-z (PMC6015043; doi:10.1038/s41426-018-0116-z)
Supplement: Supplementary file 6 — Supplementary Figure S6 [file 41426_2018_116_MOESM6_ESM.pdf]

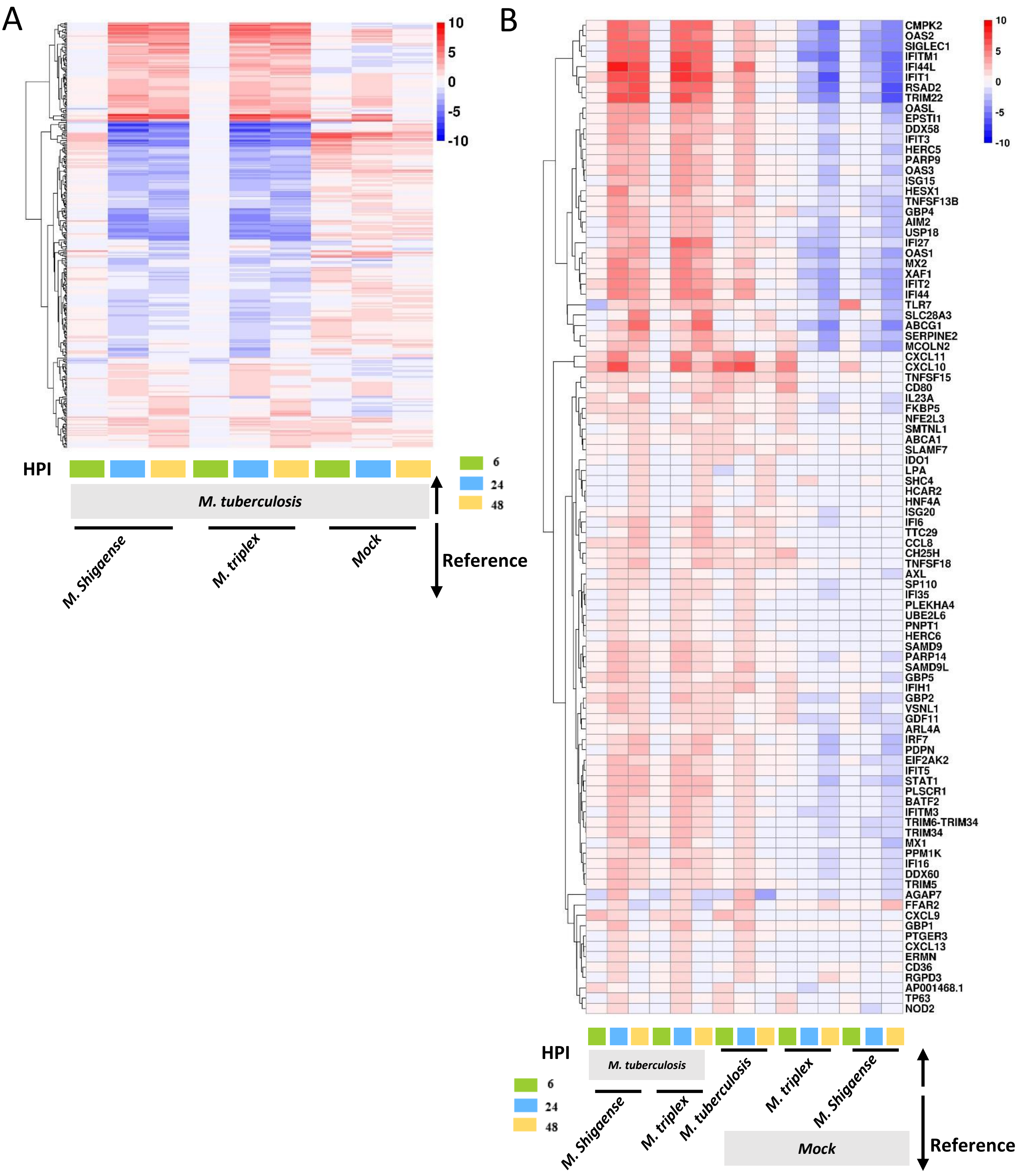

Supplementary Figure S6. Gene expression signature of *M. tuberculosis*- specific genes. (A) For all of *M. tuberculosis*- specific genes. (B) Part for up-regulated *M. tuberculosis*- specific genes, another Part for these genes of *M. tuberculosis*, *M. shigaense* and *M. triplex* compared with mock group. The colors denote log2FC of genes expression. HPI has the same meanings with Figure 5.
