# Supplementary material for: Landscape of the genome and host cell response of Mycobacterium shigaense reveals pathogenic features
Source: Emerg Microbes Infect. 2018 Jun 22;7:112. doi: 10.1038/s41426-018-0116-z (PMC6015043; doi:10.1038/s41426-018-0116-z)
Supplement: Supplementary file 7 — Supplementary Figure S7 [file 41426_2018_116_MOESM7_ESM.pdf]

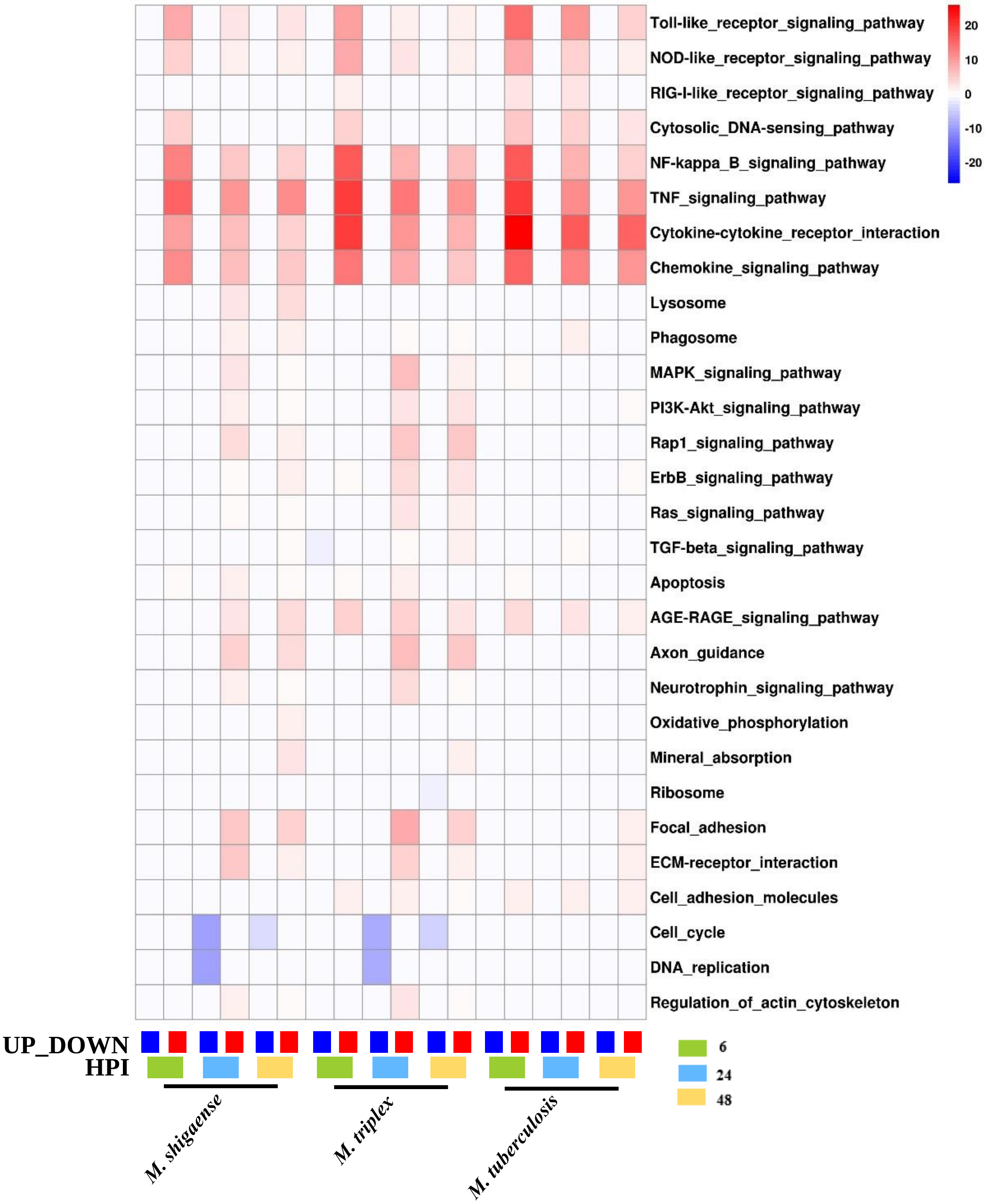

Supplementary Figure S7. KEGG pathway enrichment for *M.shigaense*, *M. triplex* and *M. tuberculosis*. Colour red means significant enrichment by up-regulated genes, and blue by down-regulated genes. Each score represents minus base 10 logarithm of enrichment p-values. The UP_DOWN row means enrichment by up-regulated genes (red) or down-regulated genes (blue).
